# Supplementary material for: The Study of the Sterilization of the Indoor Air in Hospital/Clinic Rooms by Using the Electron Wind Generator
Source: Int J Environ Res Public Health. 2019 Dec 5;16(24):4935. doi: 10.3390/ijerph16244935 (PMC6950108; doi:10.3390/ijerph16244935)
Supplement: Supplementary file 1 [file ijerph-16-04935-s001.pdf]

a)

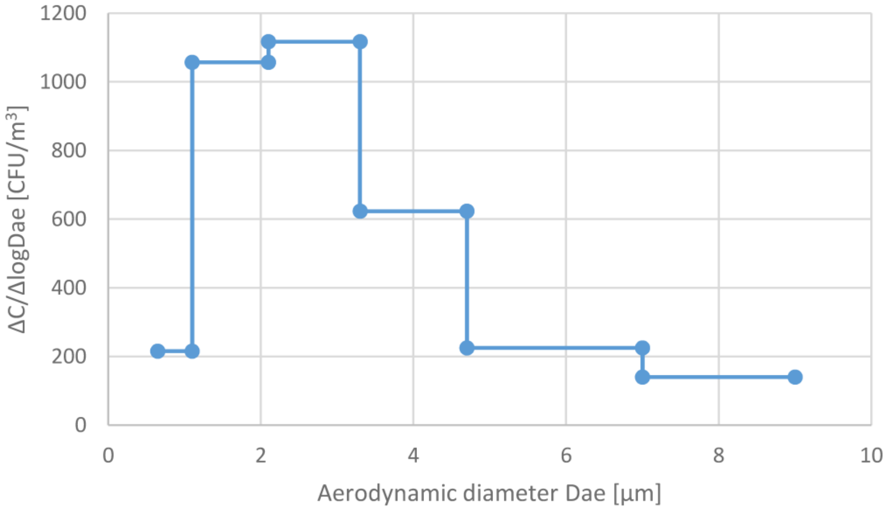

b)

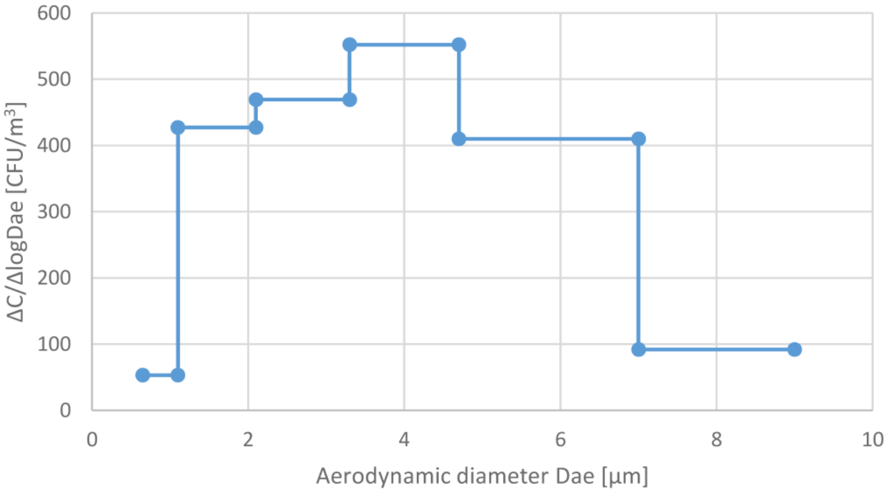

Figure S1. Size-distribution of airborne bacteria in the patient`s room (PRL) at the University Hospital in Zabrze: a) Background, b) 4 hours after start of sterilization using the EWG device.

a)

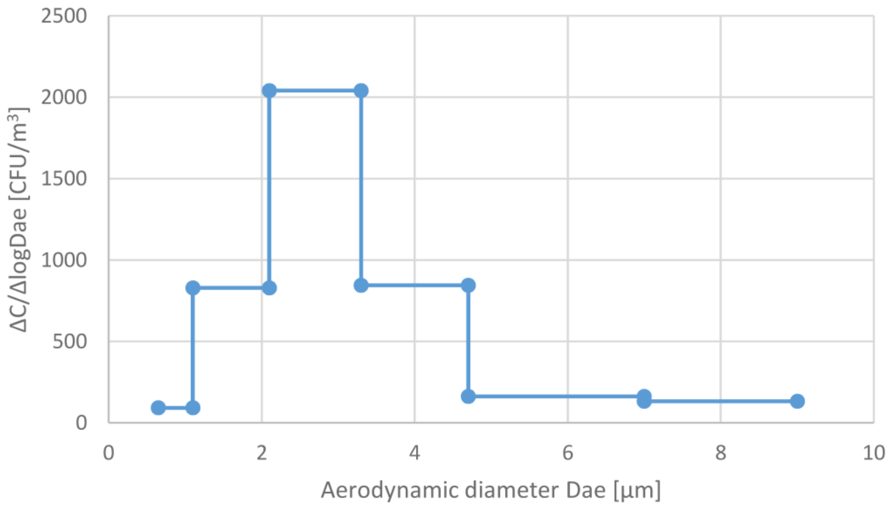

b)

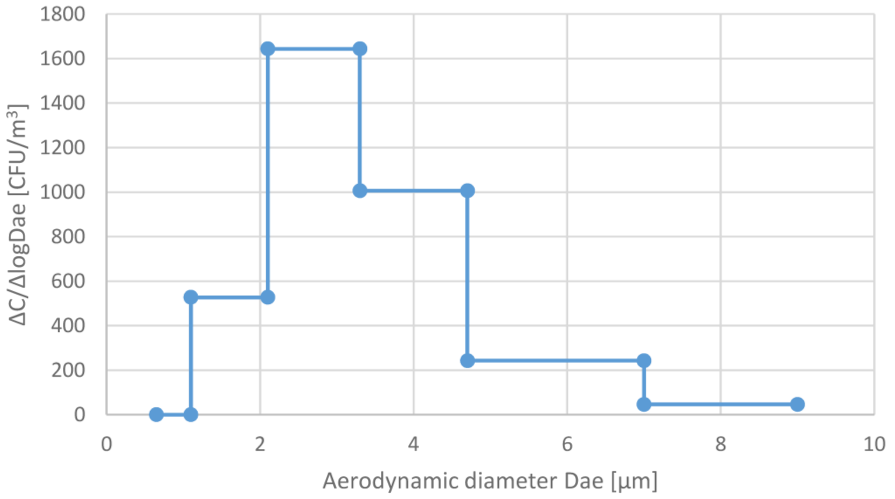

Figure S2. Size-distribution of airborne fungi in the patient`s room (PRL) at the University Hospital in Zabrze: a) Background, b) 4 hours after start of sterilization using the EWG device.

a)

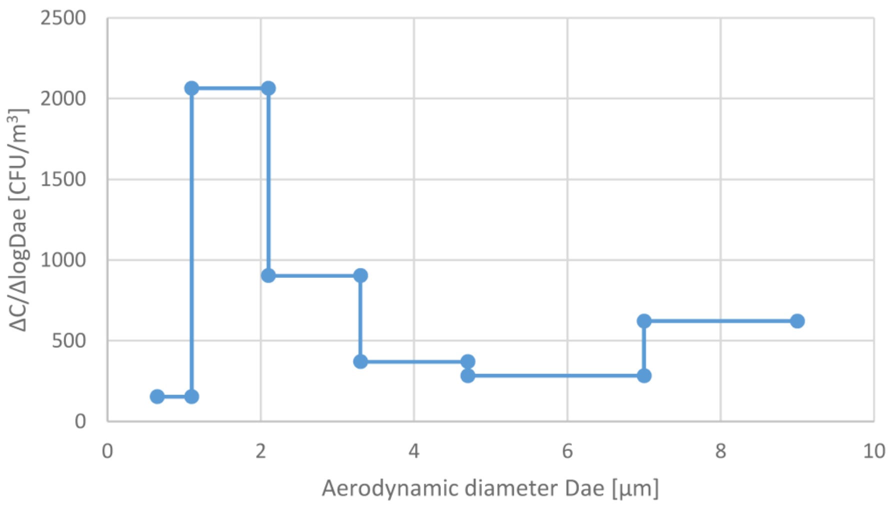

b)

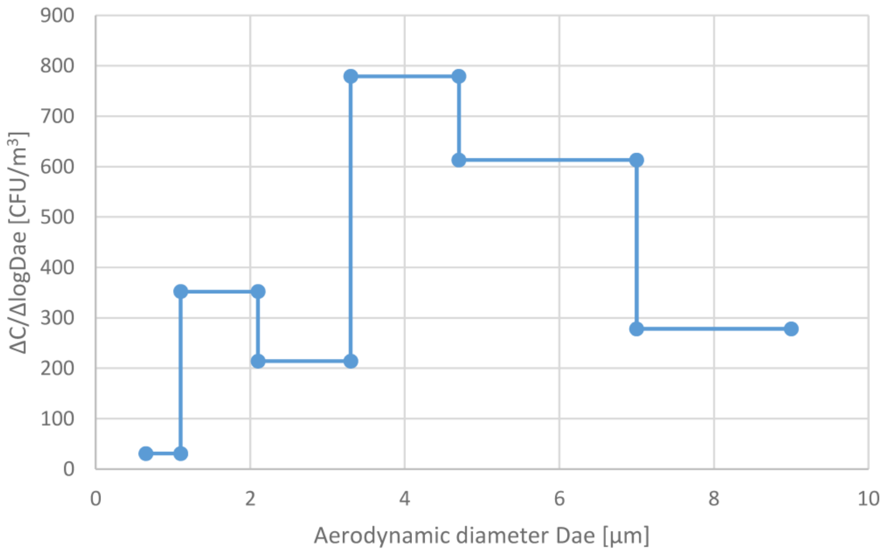

Figure S3. Size-distribution of airborne bacteria in the waiting room (WR) at the clinic in Sosnowiec: Background, b) 4 hours after start of sterilization using the EWG device.

a)

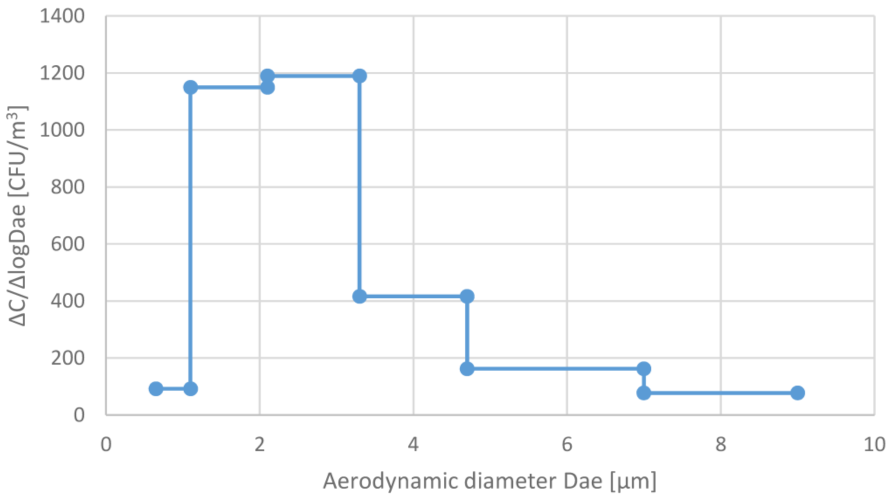

b)

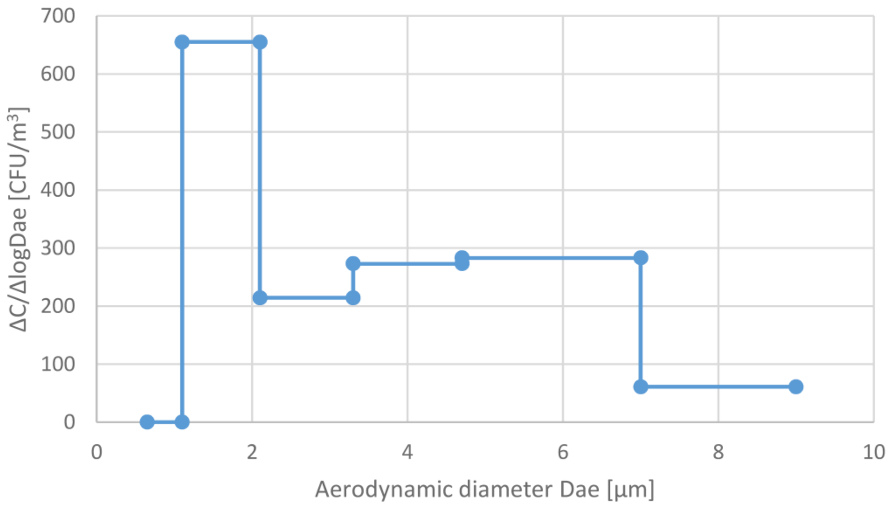

Figure S4. Size-distribution of airborne fungi in the waiting room (WR) at the clinic in Sosnowiec: a) Background, b) 4 hours after start of sterilization using the EWG device.
